# Editorial: Community series in advancing social purpose in organizations: An interdisciplinary perspective

**DOI:** 10.3389/fpsyg.2023.1116904

**Published:** 2023-01-24

**Authors:** Monica Thiel, Gabriele Giorgi, Antonio Ariza-Montes, Nicola Mucci

**Affiliations:** ^1^School of Business Administration, Asian Institute of Management, Makati, Philippines; ^2^European University of Rome, Rome, Italy; ^3^Social Matters Research Group, Universidad Loyola Andalucía, Córdoba, Spain; ^4^Department of Experimental and Clinical Medicine, University of Florence, Florence, Italy

**Keywords:** social purpose, human and social wellbeing, environment, organizations, sustainability

Social purpose is important for advancing our understanding of how to make positive impacts in society through business purpose and profit and for employees finding meaning in their lives through work. In this community series, four manuscripts highlight the importance of human and social wellbeing and what it means within an organizational psychology context. Specifically, the authors demonstrate how organizations could create innovative business models in social purpose (De Silva et al., 2021) through better management of inclusion, psychological safety, job insecurity and stress and the “United Nations Sustainable Development Goals (2015) 3, 6, 7, 8, 9, 12, 13, and 17”.

In article 1, Fantinelli et al. focus on the urgency of disability management due to stigma in social norms. The authors' findings highlight the importance of the family context and social support and confirmed previous literature about the need for job inclusiveness, a profound sense of satisfaction related to the job, strong identity, and empowerment derived from the job involvement. Hao et al. extend the knowledge sharing behavior and trust literature through identification of the psychological mechanisms and boundary conditions in trust and knowledge sharing behavior of the virtual team relationship in article 2. The authors examine the retail relationships context of work-stress-job insecurity and technological changes-job insecurity during the COVID-19 outbreak. Overall, the authors explore what makes a virtual team more efficient and vibrant and demonstrate their findings through a multilevel theoretical model that could be useful for future research.

Ghani et al. contributes to the literature in article 3 within the retail industry relationships between work-stress-job insecurity and technological changes-job insecurity. Specifically, the authors examine when retail store sales fell from positive growth of 3.11% to a negative growth of 0.31% during the COVID-19 outbreak in 2019–2020. The authors findings indicate insecurity increases when there are increases in technological changes. Hence, training and education is crucial to help employees to cope with new technology that is introduced when employees are working online. In article 4, Wang et al. examine EMS adopter and non-EMS adopter performance in companies as independent variables through dependent variables. The variables include Environmental Performance, Health and Safety, Employee Satisfaction, Operational Improvement, and Competitive Advantage. The authors' study findings confirm the likelihood of improved environmental and social performance from EMS companies when the companies identify their environmental issues more efficiently than non-EMS companies.

In conclusion, the authors remind us that social purpose can build long-term organizational success through social purpose. Moreover, the important contributions from the authors in the Research Topic help us to rediscover purpose (Hollensbe et al., 2014) in organizations.

## Author contributions

All authors listed have made a substantial, direct, and intellectual contribution to the work and approved it for publication.

## References

[B1] De Silva M. Al-Tabbaa O. Khan Z. (2021). Business model innovation by international social purpose organizations: the role of dynamic capabilities. J. Bus. Res. 125, 733–749. 10.1016/j.jbusres.2019.12.030

[B2] Hollensbe E. Wookey C. Hickey L. George G. Nichols V. (2014). Organizations with purpose: from the editors. Acad. Manag. J. 57, 1227–1234. 10.5465/amj.2014.4005

[B3] United Nations Sustainable Development Goals. (2015). The 17 Goals. Department of Economic and Social Affairs. Retrieved from: https://sdgs.un.org/goals

